# Breast Feeding, Parity and Breast Cancer Subtypes in a Spanish Cohort

**DOI:** 10.1371/journal.pone.0040543

**Published:** 2012-07-11

**Authors:** Carmen M. Redondo, Manuela Gago-Domínguez, Sara Miranda Ponte, Manuel Enguix Castelo, Xuejuan Jiang, Ana Alonso García, Maite Peña Fernández, María Ausencia Tomé, Máximo Fraga, Francisco Gude, María Elena Martínez, Víctor Muñoz Garzón, Ángel Carracedo, J. Esteban Castelao

**Affiliations:** 1 Oncology and Genetics Unit, Genomic Medicine Group, Complejo Hospitalario Universitario de Vigo, Vigo, Spain; 2 Genomic Medicine Group, Galician Foundation of Genomic Medicine, Complejo Hospitalario Universitario de Santiago, IDIS, Santiago de Compostela, Spain; 3 Radiotherapy Department, Complejo Hospitalario Universitario de Vigo, Vigo, Spain; 4 Ophtalmology and Preventive Medicine Department, University of Southern California, Los Angeles, California, United States of America; 5 Radiotherapy Department, Hospital Universitario Central de Asturias, Oviedo, Spain; 6 Ginecology Department, Complejo Hospitalario Universitario de Santiago, Santiago de Compostela, Spain; 7 Endocrinology Department, Complejo Hospitalario Universitario de Santiago, Santiago de Compostela, Spain; 8 Pathology Department, Complejo Hospitalario Universitario de Santiago, Santiago de Compostela, Spain; 9 Clinical Epidemiology Unit, Complejo Hospitalario Universitario de Santiago, Santiago de Compostela, Spain; 10 University of California San Diego, Moores Cancer Center, San Diego, California, United Sates of America; Ohio State University Medical Center, United States of America

## Abstract

**Background:**

Differences in the incidence and outcome of breast cancer among Hispanic women compared with white women are well documented and are likely explained by ethnic differences in genetic composition, lifestyle, or environmental exposures.

**Methodolgy/Principal Findings:**

A population-based study was conducted in Galicia, Spain. A total of 510 women diagnosed with operable invasive breast cancer between 1997 and 2010 participated in the study. Data on demographics, breast cancer risk factors, and clinico-pathological characteristics were collected. The different breast cancer tumor subtypes were compared on their clinico-pathological characteristics and risk factor profiles, particularly reproductive variables and breastfeeding. Among the 501 breast cancer patients (with known ER and PR receptors), 85% were ER+/PR+ and 15% were ER-&PR-. Among the 405 breast cancer with known ER, PR and HER2 status, 71% were ER+/PR+/HER2- (luminal A), 14% were ER+/PR+/HER2+ (luminal B), 10% were ER−/PR−/HER2- (triple negative breast cancer, TNBC), and 5% were ER−/PR−/HER2+ (non-luminal). A lifetime breastfeeding period equal to or longer than 7 months was less frequent in case patients with TNBC (OR = 0.25, 95% CI = 0.08–0.68) compared to luminal A breast cancers. Both a low (2 or fewer pregnancies) and a high (3–4 pregnancies) number of pregnancies combined with a long breastfeeding period were associated with reduced odds of TNBC compared with luminal A breast cancer, although the association seemed to be slightly more pronounced among women with a low number of pregnancies (OR = 0.09, 95% CI = 0.005–0.54).

**Conclusions/Significance:**

In case-case analyses with the luminal A cases as the reference group, we observed a lower proportion of TNBC among women who breastfed 7 or more months. The combination of longer breastfeeding duration and lower parity seemed to further reduce the odds of having a TNBC compared to a luminal A breast cancer.

## Introduction

In the US, breast cancer impacts each racial group differently [1]–[4]. Compared with non-Hispanic White (NHW) women, Hispanic women have a lower incidence rate of breast cancer, however, once diagnosed with this disease they are more likely to die from it [5]. Studies [6], [7] have found that despite equal access to health care services, differences persist in the presentation of Hispanic women with breast cancer compared with NHW women, suggesting a biologic basis for the racial/ethnic differences. The potential biological differences among breast cancers may result from racial/ethnic differences in genetic composition, lifestyles or environmental exposures [7].

It has been reported that women diagnosed with estrogen receptor-positive (ER+)/progesterone receptor-positive (PR+) tumors are more responsive to hormonal treatment and have a better prognosis than those diagnosed with estrogen receptor-negative (ER-)/progesterone receptor negative (PR-) tumors, suggesting etiologic heterogeneity of hormone-receptor defined subtypes of breast cancer [8], [9]. Consistently, disparate risk factor profiles for breast cancer according to ER and PR status have been reported [1], [10]. In general, Hispanic patients with breast cancer tend to have ER- tumors more disproportionately than NHW women although the difference was not as great as that seen between black and NHW women [11], [12].

In this study, we describe the characteristics of breast cancer subtypes defined by ER, PR and HER2 receptor status and assessed the associations between reproductive factors and breastfeeding and tumor subtypes in a case series of female breast cancer patients from Galicia, a region located in the northwest part of Spain, whose history has been defined by mass emigration to Latin America [13]. Because this region has been the European state with one of the highest emigration to Latin America in the 1800s and 1900s, its population could be, at least partially, a contributor of the European ancestry to Hispanics in the US. In addition, the Galician population could provide a contrast group to Hispanics from regions in the U.S. such as the San Luis Valley, Colorado in the US, many of whom self-identify as being of “Spanish origin” [14].

To our knowledge, this is in one of the first studies to explore these relationships in this population.

## Materials and Methods

### Study Population

A population-based study, which is part of the Breast Oncology Galician Network (BREOGAN) Study, was conducted in the city of Vigo, Spain within a geographically defined health region that covers aproximately 437,000 inhabitants. The study involved 510 women with operable invasive breast cancer diagnosed and treated between 1997 and 2010 at the Clinical University Hospital of Vigo (Vigo, Spain). Ethics approval for this study was obtained from the Galician Ethics and Research Committee (CEIC, Comité Ético de Investigación Clínica de Galicia) associated with the Complejo Hospitalario Universitario de Vigo from where all participants were recruited. Written informed consent was obtained for this study, which was conducted according to the Spanish law including adherence to the Helsinki Principles of 1975, as revised in 1983.

### Data Collection

Risk factor information was collected through a risk factor questionnaire adapted from the *Ella* Binational Breast Cancer Study [15]. Clinical and histopathological information was abstracted from computerized medical records by trained physicians. The following variables were recorded: lifetime breastfeeding (categorized as no breastfeeding, < mean lifetime breastfeeding duration (7 months), ≥ mean lifetime breastfeeding duration (7 months)), age at menarche (categorized as ≤13 years, 14 years, ≥15 years), age at first full-term pregnancy (categorized as ≤22 years, 23–27 years, ≥28 years), parity (categorized as never vs. ever pregnant), age at diagnosis (categorized when being the main variable of the analysis as <50 years, ≥50 years, otherwise treated as a continous variable), age at menopause (categorized as <50 years, ≥50 years), menopausal status at diagnosis (categorized as pre, peri and postmenopausal), number of pregnancies (categorized as none, 1–2, ≥3), family history (categorized as none vs. one or more first degree relatives with breast and/or ovarian cancer), ER, PR and HER2 status (categorized as positive and negative), grade (categorized as I – well differenciated -, II – moderately differenciated- and III – poorly differenciated or undiferenciated), histology type (categorized as invasive ductal carcinoma, invasive lobular carcinoma and medular carcinoma), and tumor size (categorized as ≤1 cm, >1 - <2 cm, ≥2 cm). Of the 510 women who participated in the study, 1 had unknown ER status, 8 had unknown PR status, 9 had unknown ER and PR status and 105 had unknown joint ER, PR and HER2 status. Thirty eight women had unknown grade, one had unknown histological type and 21 had unknown tumor size. Two women had unknown age at menarche and two (out of 423 parous women) had unknown lifetime breastfeeding.

#### Clinico-pathological data

Histopathological information was abstracted from computerized medical records by trained physicians. Immunohistochemistry (IHC) analyses on paraffin-embedded material have been previously performed following standard procedures in Galician hospitals to determine the status of ER and PR. In every tumor, 4-µm histological sections were cut and stained with hematoxylin and eosin for histopathological examination according to the criteria of the World Health Organization [16]. Histological grading was evaluated using the Nottingham modification of the Bloom-Richardson system [17]. IHC analysis on paraffin-embedded material was performed using a universal second antibody kit that used a peroxidase-conjugated labeleddextran polymer (EnVision®, Peroxidase/DAB; Dako, Glostrup, Denmark), with antibodies for ER (clone 6F11, dilution 1∶50, water bath; Novocastra, Newcastle-upon- Tyne, UK), PR (clone PgR 636, dilution 1∶50, water bath; Dako, Glostrup, Denmark). Negative and positive controls were concurrently run for all antibodies with satisfactory results. Cells were considered immunopositive when diffuse or dot-like nuclear staining was observed regardless of the intensity of the staining; only nuclear immunoreactivity was considered specific. The number of positive cells was counted by two different observers independently. Whenever necessary, a consensus was reached using a double-headed microscope. ER and PR were considered positive when the percent of immunostained nuclei was ≥10%.

Immunohistochemistry (IHC) analyses were performed to determine HER2 status (Dako). No immunostaining (0) or weak membrane immunostaining (1+) was considered low HER2 expression (HER2-). Strong membrane immunostaining (3+) was considered HER2 overexpression (HER2+). Moderate membrane staining (2+) samples were further analyzed using fluorescence in situ hybridization techniques; they were considered to be HER2+ if the ratio of cerb-B2/centromere 17 copy number was >2.0.

### Statistical Analyses

We classified breast tumors according to their expression of ER (n = 509), PR (n = 502) and joint status of both ER and PR expression (n = 501). For patients with data available on ER, PR, and HER2 (n = 405 case patients), we defined four tumor subtypes (ER+/HER2- or PR+/HER2- [luminal A], ER+/HER2+ or PR+/HER2+ [luminal B], ER−/PR−/HER2+ [non-luminal], and ER−/PR−/HER2-[triple negative]), shown in Table 1). Case-case analysis was conducted. Multivariate logistic regression was used to estimate odds ratios (ORs), 95% confidence intervals (CIs), and P values for associations between the different risk factors and breast cancer subtypes while simultaneously controlling for age at diagnosis, age at menarche, age at first full-term pregnancy (only in analyses restricted to parous women), menopausal status at diagnosis and family history of first degree relatives with breast and/or ovarian cancer. Outcome (dependent) variables were breast cancer subtypes defined by ER, PR, and HER2 status, and explanatory variable was the risk factor being studied at the time. P values were calculated using likelihood ratio tests.

All statistical analyses were performed using the R statistical software (R_2.13.0). All reported test significance levels (P values) were two-sided.

**Table 1:** Characteristics of the breast cancer patients included in the study (N = 510).

	N (%)
**Age at diagnosis** ± SD	54.7±12.7 years
**Age at menarche** ± SD	13.2±1.7 years
**Age at first full-term pregnancy** ± SD	24.7±4.6 years
**Parity**	
No	87 (17.1)
Yes	423 (82.9)
**Number of pregnancies** ± SD	2.3±1.0
**Breastfeeding**	
No	144 (34.2)
Yes	277 (65.8)
**Lifetime breastfeeding**	7.1±10.5 months
**Grade**	
I	99 (21.2)
II	233 (49.9)
III	135 (28.9)
**Histology type**	
Ductal Invasive	406 (79.8)
Lobulillar Invasive	50 (9.8)
Medullary	11 (2.2)
Other	42 (8.2)
**Tumor size**	2.2±1.3 cm
**ER**	
Positive	422 (82.9)
Negative	87 (17.1)
**PR**	
Positive	348 (69.3)
Negative	154 (30.7)
**ER/PR**	
ER+/PR+	337 (67.2)
ER+/PR-	78 (15.6)
ER−/PR+	10 (2.0)
ER−/PR-	76 (15.2)
**ER/PR/HER2**	
ER+/or PR+/HER2-	287 (70.9)
ER+/or PR+/HER2+	58 (14.3)
ER−/PR−/HER2+	21 (5.2)
ER−/PR−/HER2-	39 (9.6)

## Results

A total of 501 breast cancer patients with known ER and PR and 405 with known ER, PR, and HER2 were identified. Among those with known ER and PR status, 85% were ER+/PR+ and 15% were ER-&PR- and among those with data for all three markers, 71% were ER+/PR+/HER2- (luminal A), 14% were ER+/PR+/HER2+ (luminal B), 10% were ER−/PR−/HER2- (TNBC), and 5% were ER−/PR−/HER2+ (non-luminal). The age of these patients ranged from 28 to 84 years, with a mean of 54.7±12.7 years. Detailed characteristics of the study population are presented in Table 1.

Table 2 shows the associations between breast cancer risk factors and TNBC in comparison to luminal A breast cancer. TNBC phenotype was significantly associated with shorter duration of breastfeeding after adjustment for other breast cancer risk factors. A lifetime breastfeeding period equal or longer than 7 months was inversely associated with the odds of having a triple-negative tumor (versus a luminal A tumor) (OR = 0.25, 95% CI = 0.08– 0.68). Parity was more frequent in case patients with TNBC compared to luminal A breast cancers (OR = 1.81, 95% CI = 0.67–6.32). No meaningful associations were found between other reproductive or menstrual factors and TNBC.

**Table 2:** Associations between breast cancer risk factors/tumor characteristics and triple-negative breast cancer.

		Luminal A	TNBC		
		N (%)	N (%)	OR (95% CI)* Multivariate	P_LRT_
**Age at first full-term pregnancy**	≤22	85 (90.4)	9 (9.6)	1.0	
	23–27	90 (83.3)	18 (16.7)	2.22 (0.93–5.70)	
	≥28	61 (88.4)	8 (11.6)	1.38 (0.48–3.93)	0.190
**Age at menarche**	≤13	167 (86.5)	26 (13.5)	1.00	
	14	64 (88.9)	8 (11.1)	0.82 (0.33–1.85)	
	≥15	52 (91.2)	5 (8.8)	0.65 (0.21–1.68)	0.672
**Age at diagnosis**	<50	114 (85.1)	20 (14.9)	1.00	
	≥50	169 (89.9)	19 (10.1)	0.58 (0.18–1.81)	0.359
**Age at menopause**	<50	155 (88.6)	20 (11.4)	1.00	
	≥50	128 (87.1)	19 (12.9)	1.28 (0.63–2.61)	0.488
**Menopausal status**	Pre-menopausal	66 (86.8)	10 (13.2)	1.00	
	Peri-menopausal	62 (84.9)	11 (15.1)	1.25 (0.48–3.25)	
	Post-menopausal	155 (89.6)	18 (10.4)	0.99 (0.28–3.44)	0.865
**Family History**	No	226 (87.9)	31 (12.1)	1.00	
	Yes	57 (87.7)	8 (12.3)	1.09 (0.44–2.43)	0.848
**Lifetime duration of breastfeeding**	No	76 (82.6)	16 (17.4)	1.00	
	<7 months	69 (83.1)	14 (16.9)	0.91 (0.39–2.06)**	
	≥7 months	89 (94.7)	5 (5.3)	0.25 (0.08–0.68)**	0.012
**Parity**	No	47 (92.2)	4 (7.8)	1.00	
	Yes	236 (87.19)	35 (12.9)	1.81 (0.67–6.32)	0.258
	1–2	164 (86.8)	25 (13.2)	1.80 (0.65–6.38)	
	≥3	72 (87.8)	10 (12.2)	1.84 (0.56–7.23)	0.526

* Adjusted for age at diagnosis, age at menarche, menopausal status and family history except in models with any of these variables as main predictors.

** Further adjusted for age at first full-term pregnancy.

We further examined the joint effect of breastfeeding and parity and tumor subtype. Table 3 shows the association between the average lifetime duration of breastfeeding and odds of having TNBC versus luminal A breast cancer within case groups defined by parity. Among women with 2 or fewer full-term pregnancies, breastfeeding for 7 months or longer was inversely associated with the odds of having a triple-negative tumor versus a luminal A tumor (OR = 0.09, 95% CI = 0.005–0.54) after adjustment for age at diagnosis, age at menarche, age at first full-term pregnancy, menopausal status and family history; however, this finding is based on only 1 TNBC with number of full-term pregnancies 2 o lower who breastfed for 7 months or longer. Among women with 3 or more pregnancies, breastfeeding duration of 7 months or longer was also inversely associated with the odds of having a triple-negative tumor (versus luminal A tumors) although the association lacked precison (OR = 0.37, 95% CI = 0.08–1.65). No statistically significant interaction between breastfeeding and parity and odds of TNBC vs. luminal A subtype was shown (P = 0.41).

**Table 3:** Associations between parity and lifetime breastfeeding and luminal A and triple-negative breast cancer.

	Luminal A (N = 162)	TNBC (N = 35)		
	N (%)	N (%)	OR (95% CI)* Multivariate	P_LRT_
**Parity ≤2**				
No Breastfeeding	53 (82.8)	11 (17.2)	1.00	
Breastfeeding <7 months	62 (82.7)	13 (17.3)	0.92 (0.36–2.40)	
Breastfeeding ≥7 months	47 (97.9)	1 (2.1)	0.09 (0.005–0.54)	0.009
**Parity ≥3**				
No Breastfeeding	23 (82.1)	5 (17.9)	1.00	
Breastfeeding <7 months	7 (87.5)	1 (12.5)	0.88 (0.04–8.37)	
Breastfeeding ≥7 months	42 (91.3)	4 (8.7)	0.37 (0.08–1.65)	0.406

* Adjusted for age at diagnosis, age at menarche, age at first full-term pregnancy, menopausal status and family history.

No associations were shown between other breast cancer risk factors (age at menarche, age at first pregnancy, age at menopause, and family history) and the different tumor subtypes (Table S1).

Regarding tumor characteristics, high grade (OR = 47.38, 95% CI = 6.14–365.30, P<0.001) and medullar type breast cancer (OR = 35.30, 95% CI = 6.84–182.10, P<0.001), were more frequent in case patients with triple-negative tumors compared to luminal A tumors (Table S2).

## Discussion

In a population-based study of breast cancer patients from Spain, we observed that the proportion of cases with TNBC versus luminal A breast cancer was lower in women who breastfed for 7 or more months than in those who did not breastfeed. Also, compared with luminal A breast cancers, TNBCs were more common in parous vs. non-parous women. Both a low (2 or fewer pregnancies) and a high (3–4 pregnancies) number of pregnancies combined with a long breastfeeding period were associated with reduced odds of TNBC compared with luminal A breast cancer; although the association seemed to be slightly more pronounced among women with a low number of pregnancies we lacked the statistical power to detect any difference. No other associations were detected between tumor subtypes and other reproductive/lifestyle breast cancer risk factors despite the accumulating evidence favoring distinct reproductive profiles among the differing tumor subtypes [18], [19].

Several case-control and cohort studies have examined the association between parity and breastfeeding and the risk of TNBC. Although an inverse association of parity with risk of ER+ breast cancer has been found by many studies, studies of ER– breast cancer indicate a positive, risk-enhancing association with parity [18]–[21]. Studies including “intrinsic” breast cancer subtypes based on additional molecular markers such as the basal-like subtype also found high parity to be associated with an increased risk [18], [19]. In some of these studies, this association was present only among women who had never breastfed [19], [21]. Several studies have reported a lower risk of TNBC in parous women who have ever breastfed a child [22], or who breastfed for a cumulative duration of at least 4 [19], 6 [23], [24], or 12 months [25]. Only two case–case analyses have compared the TNBC subtype with the ER+/PR+ subtype and both found a strong positive association of parity with TNBC [26] or with ER−/PR−/HER2+ tumors [27]. Both studies also observed a reduced odds of TNBC (compared to non-TNBC and to luminal A breast cancer) associated with breastfeeding [26], [27], and, in one, the positive association of parity with TNBC was present only among women who had never breastfed [27]. We found some evidence in support of these findings in our study of Spanish women from Galicia. We observed reduced odds of TNBC compared to luminal A breast cancer associated with breastfeeding, and some indication that the combination of a longer breastfeeding duration and decreased parity may reduce the odds of having a TNBC compared to a luminal A breast cancer, although this finding was based on very small numbers (only one TNBC with number of full-term pregnancies 2 o lower who breastfed for 7 months or longer).

It is unclear why the inverse association between breastfeeding and TNBC tends to be more pronounced among Hispanics than NHWs [26]. In general, Hispanic patients with breast cancer tend to have ER-negative tumors more frequently than NHW women [11], [12], [28], [29]. In one study using data from 13 Surveillance, Epidemiology, and End Results cancer registries over a period from 1992 through 2004 [30], researchers analyzed age-adjusted incidence rates and trends from women older than 50 years and showed an increase in incidence rates of ER and PR-negative tumors among Hispanic women compared with NHW women (14.2% in white women compared with 17.3% in Hispanic women). They reported a relative increase in rates of TNBC in Hispanic women (from 2001 through 2004) of 26.8%, with an absolute increase from 34.6 to 43.9 [30]. Our findings that TNBC was more likely to be of a high grade, and high tumor size at diagnosis support the hypothesis that the presence or absence of ER and PR represents distinct biological entities rather than different stages in the natural history of the disease.

It is possible that genetic susceptibility to breast cancer may differ among Hispanics and other ethnicities. Major differences in gene expression between Hispanics and NHW have been detected [5]. In one such study, researchers [5], [31] hypothesized that during pregnancy, progenitor cells that coexpress GABRP (the gamma-aminobutyric acid A receptor, *GABRP,* a type A receptor that is expressed in reproductive tissues), proliferate within the breast lobules and then are lost during breastfeeding. These investigators evaluated 203 newly diagnosed invasive breast cancers and showed that higher *GABRP* gene expression was more common in younger women with a limited history of lactation after pregnancy study [31]. The same study showed that *GABRP* gene expression was higher in Hispanic women compared with white controls. This investigation suggested that *GABRP* gene expression may be associated with high-grade breast cancer in Hispanic women; although this needs additional study.

The present study was conducted in Galicia, a region located in the northwest part of Spain, whose history has been defined by mass emigration to Latin America [13]. In the United States, Hispanics are a diverse and growing community that represents 12% of the US population [32]. Hispanic ethnicity represents a culturally and genetically heterogeneous group [33]. Hispanics are basically tri-hybrid, i.e., their ancestral populations being European, African, and Native American with the European contribution usually being the highest, although this varies to a degree [34]. Because Galicia has been the European state with one of the highest emigration rates to Latin America, its population could be, at least partially, a contributor of the European ancestry to Hispanics in the US. Thus, in a *BRCA1* screening study in familial breast cancer carried out in different centers in Spain, France and the United Kingdom, the missense mutation 330 A>G was independently identified in six families, all of them with Spanish/Galician ancestors [35]. This mutation has been observed in families in diverse geographical locations (Spain, Caribbean, France, United Kingdom) who would appear to be of Spanish origin [35]. These studies are consistent with the possibility that the families studied shared a common ancestry with *BRCA1* 330 A>G being a founder mutation of Spanish/Galician origin. Similarly, the BRCA1 185delAG mutation has been identified among several apparently non-Jewish families in Spain [36]–[40], one Chilean family [41], and in several Mexican and Hispanic American cohorts [42]–[44]. The 185delAG families identified among the Spanish cohorts [36]–[40] are likely descendants of the Jewish that remained in Spain, whereas the carriers reported in the other cohorts are likely descendants of Spanish Jews who emigrated to the Americas in the late 15th century and assimilated into the larger Hispanic society [44].

Although our results are mostly in agreement with those of other studies, limitations of our study should be discussed, notably its small sample size particularly in subset analyses stratified by hormonal receptor status ER, PR or HER2. Sample sizes for the less common subtypes were limited. In addition, the breastfeeding data were based on self reported information collected years later. In general, breastfeeding history has been shown to be accurate and reliable [45], [46]. However, other authors have shown missclassification [47], although it has been found to be non-differential, i.e., to attenuate the true strength of the association between breastfeeding and the health event under study [47]. Thus, even if there was misclassification in the present study, the true association between breastfeeding and TNBC would have possibly been stronger than the observed. Another limitation relates to the fact that since breast cancer receptor status had been determined from medical records in the present study**,** there could be the possibility of potential heterogeneity in reading stains and scoring of immunohistochemistry; however this limitation would be expected to bias the study results towards the null. Finally, the case-case study design has obvious limitations [9]. A study that does not include a disease-free population does not provide a traditional risk ratio and may not provide a valid estimate of the association between a risk factor and disease; thus, the case-case OR estimated in this study cannot be interpreted as a measure of risk for the specific subtype. Furthermore, the magnitude of the association is not the magnitude of risk, but rather an indicator of the general direction of the correlation between risk factor and subtype. Thus, results from case-case analyses like ours must be validated in traditional case-control and cohort studies to asses risk and estimate the magnitude of the effect.

Our case-case design study can be particularly useful in assessing the relative correlation of established risk factors and the different tumor subtypes. We have included in our study detailed tumor marker information, such as ER, PR and Her-2 receptor status, which is needed to identify etiologic heterogeneity for established breast cancer risk factors and disease subtypes [9].

In conclusion, this analysis shows that associations between breast cancer and reproductive factors or breastfeeding vary by breast cancer tumor subtypes defined by ER, PR, and HER2 status, particularly luminal A and TNBC. These results are in concordance with emerging evidence that relationships for genetic susceptibility loci also vary by expression levels of markers in tumors [48]. Our results support the view that there may be more than one type of breast cancer from an etiological perspective, and specifically support the hypothesis that hormone receptor negative tumors may have a different etiology than hormone receptor positive tumors. Given the proposed disease heterogeneity observed in breast cancer, future large epidemiological studies will be helpful in identifying etiologic heterogeneity for the established risk factors by disease subtype. Breastfeeding, for example, may be a potential modifiable factor that may be related to the development of a specific breast cancer tumor subtype, and not to all tumor subtypes. Knowledge gained from these studies is likely to produce important information on specific risk factors by tumor subtype which would help in risk prediction models and risk reduction strategies.

## Supporting Information

Table S1 **Associations between other reproductive and lyfestyle breast cancer risk factors and different tumor subtypes.**
(DOC)

Table S2 **Associations between tumor characteristics and different tumor subtypes.**
(DOC)
