# Supplementary material for: Breast Feeding, Parity and Breast Cancer Subtypes in a Spanish Cohort
Source: PLoS One. 2012 Jul 11;7(7):e40543. doi: 10.1371/journal.pone.0040543 (PMC3394701; doi:10.1371/journal.pone.0040543)
Supplement: Table S1 — Associations between other reproductive and lyfestyle breast cancer risk factors and different tumor subtypes. (DOC) [file pone.0040543.s001.doc]

**Table S1.** Associations between other reproductive and lyfestyle breast cancer risk factors and different tumor subtypes.

|  | **Age at first full-term pregnancy** | | | | | | **Age at menarche** | | | | | **Age at menopause** | | | | | | **Family History** | | | | | | | | |
| --- | --- | --- | --- | --- | --- | --- | --- | --- | --- | --- | --- | --- | --- | --- | --- | --- | --- | --- | --- | --- | --- | --- | --- | --- | --- | --- |
| **Tumor subtypes** | | **≤ 22** | **23-27** | **≥ 28** | ≥ 28 vs ≤ 22 |  | **≤ 13** | **14** | **≥ 15** | ≥ 15 vs ≤ 13 |  | | **< 50** | **≥ 50** | ≥ 50 vs < 50 | |  | | **No** | | **Yes** | | Yes vs No | |  |  |
|  | | N (%) | N (%) | N (%) | OR (95% CI)* | P_LRT_ | N (%) | N (%) | N (%) | OR (95% CI)* | P_LRT_ | | N (%) | N (%) | OR (95% CI)* | | P_LRT_ | | N (%) | | N (%) | | OR (95% CI)* | | P_LRT_ |  |
|  | |  |  |  |  |  |  |  |  |  |  | |  |  |  | |  | |  | |  | |  | |  |  |
| **ER+** | | 129 (37.3) | 131 (37.9) | 86 (24.9) | 1.00 |  | 244 (58.4) | 95 (22.7) | 79 (18.9) | 1.00 |  | | 231 (55.3) | 187 (44.7) | 1.00 | |  | | 335 (80.1) | | 83 (19.9) | | 1.00 | |  |  |
| **ER-** | | 21 (28.4) | 35 (47.3) | 18 (24.3) | 1.29 (0.64-2.58) | 0.188 | 56 (64.4) | 15 (17.2) | 16 (18.4) | 0.95 (0.50-1.73) | 0.519 | | 41 (47.1) | 46 (52.9) | 1.59 (0.98-2.60) | | 0.061 | | 71 (81.6) | | 16 (18.4) | | 0.97 (0.52-1.73) | | 0.937 |  |
| **PR+** | | 106 (37.9) | 106 (37.9) | 68 (24.3) | 1.00 |  | 199 (57.5) | 81 (23.4) | 66 (19.1) | 1.00 |  | | 186 (53.8) | 160 (46.2) | 1.00 | |  | | 279 (80.6) | | 67 (19.4) | | 1.00 | |  |  |
| **PR-** | | 41 (30.8) | 57 (42.9) | 35 (26.3) | 1.39 (0.80-2.41) | 0.250 | 97 (63.8) | 27 (17.8) | 28 (18.4) | 0.89 (0.53-1.47) | 0.347 | | 81 (53.3) | 71 (46.7) | 1.07 (0.72-1.59) | | 0.738 | | 121 (79.6) | | 31 (20.4) | | 1.11 (0.68-1.78) | | 0.675 |  |
| **ER+/PR+** | | 103 (38.1) | 102 (37.8) | 65 (24.1) | 1.00 |  | 191 (57.0) | 80 (23.9) | 64 (19.1) | 1.00 |  | | 183 (54.6) | 152 (45.4) | 1.00 | |  | | 271 (80.9) | | 64 (19.1) | | 1.00 | |  |  |
| **ER+/PR-** | | 24 (34.8) | 26 (37.7) | 19 (27.5) | 1.31 (0.66-2.61) |  | 49 (64.5) | 13 (17.1) | 14 (18.4) | 0.83 (0.43-1.61) |  | | 44 (57.9) | 32 (42.1) | 0.87 (0.51-1.46) | |  | | 58 (76.3) | | 18 (23.7) | | 1.34 (0.73-2.44) | |  |  |
| **ER-/PR+** | | 3 (33.3) | 4 (44.4) | 2 (22.2) | 0.99 (0.15-6.31) |  | 8 (80.0) | 1 (10.0) | 1 (10.0) | 0.38 (0.05-3.17) |  | | 3 (30.0) | 7 (70.0) | 3.38 (0.82-13.92) | |  | | 7 (70.0) | | 3 (30.0) | | 2.14 (0.53-8.65) | |  |  |
| **ER-/PR-** | | 17 (26.6) | 31 (48.4) | 16 (25.0) | 1.53 (0.72-3.28) | 0.557 | 48 (63.2) | 14 (18.4) | 14 (18.4) | 0.93 (0.48-1.81) | 0.628 | | 37 (48.7) | 39 (51.3) | 1.43 (0.85-2.42) | | 0.144 | | 63 (82.9) | | 13 (17.1) | | 0.93 (0.48-1.82) | | 0.582 |  |
| **ER+ /or PR+ /Her2-** | | 85 (36.1) | 90 (38.1) | 61 (25.8) | 1.00 |  | 167 (59.0) | 64 (22.6) | 52 (18.4) | 1.00 |  | | 155 (54.6) | 128 (45.4) | 1.00 | |  | | 226 (79.9) | | 57 (20.1) | | 1.00 | |  |  |
| **ER+ /or PR+ /Her2+** | | 18 (39.1) | 15 (32.6) | 13 (28.3) | 0.96 (0.43-2.13) |  | 33 (56.9) | 14 (24.1) | 11 (19.0) | 1.19 (0.56-2.56) |  | | 35 (60.3) | 23 (39.7) | 0.91 (0.49-1.67) | |  | | 50 (86.2) | | 8 (13.8) | | 0.65 (0.29-1.47) | |  |  |
| **ER-/PR-/Her2+** | | 4 (25.0) | 6 (37.5) | 6 (37.5) | 1.90 (0.50-7.19) |  | 10 (47.6) | 3 (14.3) | 8 (38.1) | 2.84 (1.04-7.75) |  | | 10 (47.6) | 11 (52.4) | 1.66 (0.65-4.24) | |  | | 17 (81.0) | | 4 (19.0) | | 0.97 (0.31-3.04) | |  |  |
| **ER-/PR-/Her2-** | | 9 (25.7) | 18 (51.4) | 8 (22.9) | 1.31 (0.47-3.66) | 0.534 | 26 (66.7) | 8 (20.5) | 5 (12.8) | 0.64 (0.23-1.77) | 0.394 | | 20 (51.3) | 19 (48.7) | 1.28 (0.63-2.61) | | 0.632 | | 31 (79.5) | | 8 (10.5) | | 1.07 (0.46-2.49) | | 0.744 |  |
|  | |  |  |  |  |  |  |  |  |  |  | |  |  |  |  | | | |  | |  | |  |  |  |

* Adjusted for age at diagnosis, age at menarche, menopausal status and family history except in models with any of these variables as main predictors.
