# Supplementary material for: Breast Feeding, Parity and Breast Cancer Subtypes in a Spanish Cohort
Source: PLoS One. 2012 Jul 11;7(7):e40543. doi: 10.1371/journal.pone.0040543 (PMC3394701; doi:10.1371/journal.pone.0040543)
Supplement: Table S2 — Associations between tumor characteristics and different tumor subtypes. (DOC) [file pone.0040543.s002.doc]

**Table S2.** Associations between tumor characteristics and different tumor subtypes.

|  | **Grade** | | | | | **Histology Type** | | | | | | **Tumor size** | | | | |
| --- | --- | --- | --- | --- | --- | --- | --- | --- | --- | --- | --- | --- | --- | --- | --- | --- |
| **Tumor subtypes** | **I** | **II** | **III** | **III vs I** |  | **DI** | **LI** | **Medullary** | **LI vs DI** | **Medullary vs DI** |  | **≤ 1** | **>1 - < 2** | **≥ 2** | **≥ 2 vs ≤ 1** |  |
|  | N (%) | N (%) | N (%) | OR (95% CI)* | P_LRT_^*^ | N (%) | N (%) | N (%) | OR (95% CI)* | OR (95% CI)* | P_LRT_^*^ | N (%) | N (%) | N (%) | OR (95% CI)* | P_LRT_^*^ |
|  |  |  |  |  |  |  |  |  |  |  |  |  |  |  |  |  |
| **ER+** | 92 (23.8) | 213 (55.0) | 82 (21.2) | 1.00 |  | 331 (86.9) | 48 (12.6) | 2 (0.5) | 1.00 | 1.00 |  | 60 (14.9) | 149 (37.0) | 194 (48.1) | 1.00 |  |
| **ER-** | 5 (6.7) | 17 (22.7) | 53 (70.7) | 12.08 (5.00-36.16) | < 0.001 | 71 (87.7) | 1 (1.2) | 9 (11.1) | 0.10 (0.01-0.48) | 20.28 (5.04-135.66) | < 0.001 | 9 (11.1) | 21 (25.9) | 51 (63.0) | 1.79 (0.86-4.10) | 0.048 |
| **PR+** | 81 (25.0) | 178 (54.9) | 65 (20.1) | 1.00 |  | 280 (88.1) | 36 (11.3) | 2 (0.6) | 1.00 | 1.00 |  | 53 (15.8) | 133 (39.7) | 149 (44.5) | 1.00 |  |
| **PR-** | 15 (11.2) | 51 (38.1) | 68 (50.7) | 5.75 (3.06-11.39) | < 0.001 | 118 (84.9) | 12 (8.6) | 9 (6.5) | 0.81 (0.39-1.59) | 10.40 (2.61-69.13) | 0.002 | 17 (11.9) | 35 (24.5) | 91 (63.6) | 1.95 (1.08-3.68) | < 0.001 |
| **ER+/PR+** | 78 (24.9) | 174 (55.6) | 61 (19.5) | 1.00 |  | 271 (88.3) | 35 (11.4) | 1 (0.3) | 1.00 | 1.00 |  | 49 (15.1) | 129 (39.8) | 146 (45.1) | 1.00 |  |
| **ER+/PR-** | 13 (18.8) | 37 (53.6) | 19 (27.5) | 1.88 (0.85-4.14) |  | 55 (80.9) | 12 (17.6) | 1 (1.5) | 1.71 (0.82-3.53) | 4.81 (0.29-78.33) |  | 11 (15.3) | 18 (25.0) | 43 (59.7) | 1.35 (0.64-2.85) |  |
| **ER-/PR+** | 3 (30.0) | 3 (30.0) | 4 (40.0) | 1.61 (0.34-7.69) |  | 8 (80.0) | 1 (10.0) | 1 (10.0) | 1.07 (0.13-9.07) | 30.91 (1.71-558.65) |  | 3 (30.0) | 4 (40.0) | 3 (30.0) | 0.34 (0.06-1.77) |  |
| **ER-/PR-** | 2 (3.1) | 14 (21.5) | 49 (75.4) | 32.15 (7.48-138.21) | < 0.001 | 63 (88.7) | 0 (0.0) | 8 (11.3) | 2.63 exp -08 (0-∞) | 33.70 (4.11-276.35) | < 0.001 | 6 (8.5) | 17 (23.9) | 48 (67.6) | 2.76 (1.11-6.89) | 0.004 |
| **ER+ /or PR+ /Her2-** | 74 (27.7) | 150 (56.2) | 43 (16.1) | 1.00 |  | 232 (85.9) | 36 (13.3) | 2 (0.7) | 1.00 | 1.00 |  | 38 (13.8) | 108 (39.3) | 129 (46.9) | 1.00 |  |
| **ER+ /or PR+ /Her2+** | 5 (8.9) | 24 (42.9) | 27 (48.2) | 9.51 (3.37-26.84) |  | 50 (96.2) | 2 (3.8) | 0 (0.0) | 0.27 (0.06-1.18) | 6.45 exp -08 (0-∞) |  | 7 (12.5) | 15 (26.8) | 34 (60.7) | 1.40 (0.57-3.46) |  |
| **ER-/PR-/Her2+** | 0 (0.0) | 6 (28.6) | 15 (71.4) | 1.24 exp 08 (0-∞) |  | 19 (100.0) | 0 (0.0) | 0 (0.0) | 3.51 exp -08 (0-∞) | 1.47 exp -07 (0-∞) |  | 1 (5.3) | 1 (5.3) | 17 (89.5) | 5.66 (0.71-45.32) |  |
| **ER-/PR-/Her2-** | 1 (3.1) | 6 (18.8) | 25 (78.1) | 47.38 (6.14-365.30) | < 0.001 | 30 (78.9) | 0 (0.0) | 8 (21.1) | 1.92 exp -08 (0-∞) | 35.30 (6.84-182.10) | < 0.001 | 3 (8.1) | 11 (9.7) | 23 (62.2) | 2.27 (0.64-8.08) | 0.003 |
|  |  |  |  |  |  |  |  |  |  |  |  |  |  |  |  |  |

* Adjusted for age at diagnosis, age at menarche, menopausal status and family history.
